# Anecdote of Thomas Paine

**Published:** 1863-03

**Authors:** 


### ANECDOTE OF THOMAS PAINE.

One night, in 1803, when Paine boarded with Carver, I stept into his room while he was preaching his doctrines to eight or ten journeymen mechanics. When he ceased, I said to him:

"Mr. Paine, the first night I slept on shore in America was at No. 8 Dutch street, New York, in an open garret, on the 17th of June, 1794. The night was hot. I laid on my ship's mattress on the floor. At midnight the lightning flashed, the thunder rolled, the wind descended, the flood came, and beat on that shingle roof. I knew not what it meant. We had no shingles in Scotland. I was in bodily fear. Sleep fled from mine eyes. I arose at four, A. M., headache, heartache, and spirits sunk down to my heels. To kill time until the people were astir below, I opened my box of books to see if they were mildewed; they had been fourteen weeks in the hold of the vessel. On the top lay a small pocket Bible. It was placed there by my pious father. I opened the Book; my eye lighted on the third chapter of Proverbs. I read the chapter twice. I was astonished. My spirits revived—my pains fled. I grasped my wrought-nail hammer, and went forth with a stout heart to earn my first cent in America, resolving to take that chapter for my pilot, and the sixth verse for my chart. Having the Bible in my pocket, I read him the chapter.

"Now, said I, Mr. Paine, the whole host of French philosophers with Voltaire at their head, and thyself, with your 'Age of Reason,' and your book of 'Common Sense,' never wrote a line to teach a boy like myself (who, before going on board the ship which carried him from his country, had never been twenty miles from his father's door), how to behave in the world, and how to shun the path of the destroyer. But every verse in this chapter is a map, and the sixth verse a guide post, and no traveler, though a fool, can err." He looked earnestly in my face as I spoke, then laying his hand on my shoulder, said, "Friend Grant, thou art a young enthusiast." His father belonged to the Society of Friends.

Thus wrote Grant Thorburn to us not long ago; both he and Thomas Paine have now passed away to give in their great account; Paine in 1809; Thorburn fifty-four years later, in 1863. Paine was a drunkard, a blasphemer, a hater of his kind, and his last hours found him indulging in bitter reflections against the associates and friends of his later years. Thorburn lived temperately, industriously, and religiously; his heart literally overflowed with human kindness, and agreeable things are said of him from all parts of the country. A thousand editors will unite in recording, "Peace be to thy memory, Grant!" and will tenderly cherish the many pleasant thoughts which gather around his name; for he was a kind-hearted and cheery old man.

It may be useful and instructive, especially to the young, to contrast the manner of the ending of these two men. Stephen Grellot, a Quaker, (Friend,) lived in the same village with Paine, Greenwich, then a suburb of New-York, and thus speaks of the last days of the author of the *Age of Reason*:

"I may not omit recording here the death of Thomas Paine, A few days previous to my leaving home on my last religious visit, on hearing that he was ill, and in a very destitute condition, I went to see him, and found him in a wretched state; for he had been so neglected and forsaken by his pretended friends, that the common attentions to a sick man had been withheld from him. The skin of his body was in some places worn off, which greatly increased his sufferings. Something that had passed between us had made such an impression upon him that he sent for me, and on being told that I was gone from home he sent for another 'Friend.' This induced a valuable young friend, (Mary Rascoe,) who had resided in my family, frequently to go and take him some little refreshment suitable for an invalid. Once, when she was there, three of his deistical companions came to the door, and in a loud, unfeeling manner, said, 'Tom Paine, it is said you are turning Christian, but we hope you will die as you have lived'—and then went away; on which, turning to Mary Rascoe, he said: 'You see what miserable comforters they are.'

"Once he asked her if she had ever read any of his writings, and on being told that she had read but very little of them, he inquired what she thought of them, adding, 'from such a one as you I expect a correct answer.' She told him that when very young his *Age of Reason* was put into her hands, but the more she read in it the more dark and distressed she felt, and she threw the book into the fire. 'I wish all had done as you,' he replied; 'for if the devil has ever had any agency in any work, he has had it in my writing that book.' When going to carry him some refreshment, she repeatedly heard him uttering the language, 'O Lord! Lord God!' or 'Lord Jesus! have mercy on me!'

"It is well known that during his last illness he wrote a good deal: this his nurse told me; but there is a total secrecy as to what has become of his writings."

Of Thorburn, the New-York *Baptist Examiner* for January 29th, 1863, says:

"Grant Thorburn's life had many lessons for the people of the present age. He was a living epitome of all the prudential virtues—honest, truthful, kirk-going, economical, temperate. It was his boast—nor was his boasting vain—that he never drank a glass of spirits in his life, nor, except under rare and dire necessity, was out of bed after nine at night, or in it after five in the morning. He eschewed all dainty living, idle companions, and profane conversation. He married early, and more than once, and was wont to wax very eloquent in praise of Franklin's celebrated advice to the young in this regard. Such a man could not fail to live long and happily, and to die loved and regretted. May he rest sweetly, for he had a gentle heart, and never wronged a living being. 'Grant Thorburn, Seedsman and Florist'—the eye still sees the sign, and one pauses again, in memory, to take the brave little Scotchman's nervous hand, and to reciprocate his quick, hearty greeting, as he *trotted* from one to another of his crowd of customers—every one a friend."

And the *Rochester Democrat:*

"Mr. Thorburn had considerable literary taste, and wrote statedly for the daily and weekly press. Some of his sketches were of deep interest, and a volume of them was afterward published, from which, in our early days, we read with no little delight. What, however, identified his name with the literature of the day was the fact that John Galt, the Scotch novelist, made him the hero of one of his novels, under the title of *Lawrie Todd.* This at once gave him a pleasant distinction on both sides of the Atlantic, and he enjoyed it so much that he frequently signed that appellation to his published pieces. In addition to this, a Scottish lady, (we think it was Mrs. Grant of Loggan,) paid him the tribute of a handsome notice in a volume of American travels. Mr. Thorburn subsequently visited England and Scotland, and was received with much attention, and on his return published a spicy volume on the scenes and the people he had met. He was too much an American to admire the style of society and thinking prevailing in the kingdom, although the purity of Scotch manners will always be admirable.

"Mr. Thorburn was, through life, a staunch Presbyterian, and having attended the ministry of John M. Mason, could boast of having enjoyed the finest pulpit eloquence in America. Once, at an early day, he went to Boston, where he attended a fashionable church, whose opera-music and general clap-trap was in such contrast with the grand simplicity of Dr. Mason, that, as he says, he thought himself in a theater. But things have changed since then, and simplicity is the exception now.

"Mr. Thorburn outlived all his generation; the fashion of the world had all passed away before he left it. He lived to see streets and people changed throughout, and a new city built up where once cattle pastured and boys went summering. He lived to see his pastor wear out to a state of imbecility, and from a giant become once more a child. He lived to see his children in the fourth generation, to whom he bequeaths a good name and a good example. Who will not say that this was well done for the poor friendless young emigrant of three-score and ten years ago?"

The New-York *Observer* instructively says, that "Thorburn was born at Delkeith, Scotland, February 18th, 1773; his mother died when he was only two years and a half old, leaving her son to the tender mercies of a careless nurse, under whose negligence young Thorburn grew up in a dwarfish condition—a weakly, delicate child, without proper clothing or nourishment.

"At the age of eight years Thorburn was placed under the treatment of a gipsy female doctor, known by the extraordinary soubriquet of 'Luk-a'-Things.' This half magician and half beggar had a contempt for ordinary drugs and drugging, and therefore, instead of giving 'castor-oil for an obstacle in the stomach,' she prescribed for the little patient plenty of fresh air and exercise, which had such a good effect that Thorburn soon gained strength and spirit, and was able in a short time to walk and run with tolerable activity. The zeal of emulation thereupon began to animate him, and it became his study in every undertaking to surpass his comrades, which he generally succeeded in doing. He applied himself with earnestness to his father's business of nail-making, and soon made himself so expert that none of his fellow-workmen could approach him. It is said of him that in one day he manufactured with his own hands *three thousand two hundred and twenty-two nails* between the hours of six o'clock in the morning and nine o'clock in the evening.

"After retiring from business he went to reside at Astoria, and subsequently (for the last eight years or more) changed his residence to New-Haven, Connecticut. In mind and body he was remarkably vigorous, up to almost the very hour of his death. He was never really sick for the last forty years, and may be said to have died literally of old age.

"It was his custom to indulge in the occupation of sawing wood every day since his residence in New-Haven, by way of exercise. This operation was performed in a shed situated in a yard attached to his dwelling. When the weather was unpropitious for indulging in this species of gymnastics, the wood and saw had to be brought into the house, and the old man would set to work with great zest at his cutting, scattering sawdust plentifully on the carpet. On Tuesday last he gave the first signs of approaching dissolution. He was in his shed, as usual, sawing, when a weakness came over him, and he went off in a fainting-fit. The doctor was summoned in all haste, and applied the necessary remedies. The patient was enjoined to remain quietly in bed, while notice was sent to his connections to attend around the death-bed of their venerable relative. To remain in bed, however, was impossible for Thorburn. He must get up, and up he got on Wednesday morning, took breakfast, and went off as usual for the wood-cutting, in spite of all remonstrances. This was about nine o'clock in the morning. The next that was seen of him he was stretched on his face in the wood-shed, with life entirely extinct. Thus ended the eventful life of Grant Thorburn."

Thorburn lived temperately, religiously, cheerily, and long; and the living follow him to his grave with many pleasant memories. Paine lived the life of a sensualist and an infidel; he died in neglect and remorse, and the curses of many, whom his writings have misled and corrupted, have already followed him on to the judgment.

